# The Safety Profile of a Portfolio of Hyaluronic Acid-Based Soft Tissue Fillers Manufactured Using MACRO Technology: A Systematic Review of Clinical Evidence

**DOI:** 10.3390/life16010110

**Published:** 2026-01-13

**Authors:** Konstantin Frank, Said Hilton, Martina Kerscher, Doris Grablowitz, Daisy Kopera, Monika Sulovsky, Leonid Kursinov

**Affiliations:** 1 Center of Plastic, Aesthetic, Hand and Reconstructive Surgery, University Hospital Regensburg, Franz-Josef-Strauß-Allee 11, 93053 Regensburg, Germany; 2 Medical Skin Center Dr. Hilton & Partners, 40212 Düsseldorf, Germany; hilton@dr-hilton.de; 3 Division of Cosmetic Science, Department of Biochemistry and Molecular Biology, University of Hamburg, 20148 Hamburg, Germany; 4 MÄZ WIEN, Medizinisch Ästhetisches Zentrum Wien, Seilerstätte 7, 1010 Wien, Austria; 5 Department of Dermatology and Venereology, Medical University of Graz, 8010 Graz, Austria; 6 Yuvell—Home of Aesthetics, Weih, 1010 Vienna, Austria; 7 Croma-Pharma GmbH, 2100 Leobendorf, Austria; leonid.kursinov@croma.at

**Keywords:** hyaluronic acid fillers, dermal fillers, soft tissue augmentation, safety profile, adverse events, biocompatibility, aesthetic medicine, crosslinking technology, systematic review

## Abstract

Hyaluronic acid (HA)-based fillers are widely used in aesthetic dermatology for their biocompatibility, reversibility, and safety; however, adverse events (AEs) may occur. This review evaluated the safety profile, focusing on short- and long-term AEs, of HA fillers manufactured with MACRO (MAtrix CROsslinking) Core Technology, encompassing both current saypha and former Princess products. A systematic PubMed search identified prospective clinical trials assessing safety outcomes following facial aesthetic use of these fillers. Eleven studies including 947 patients met the inclusion criteria. The most common short-term AEs were transient swelling, injection site pain, and bruising, which were predominantly mild to moderate and resolved within two weeks. Severe or serious treatment-related events were rare, with only one reported across all studies. Long-term AEs, such as delayed-onset nodules or inflammatory reactions, were infrequent and mild, with no granulomas, hypersensitivity responses, or vascular complications observed. Safety outcomes were consistent across formulations and between the legacy Princess and current saypha products. Overall, the saypha HA filler portfolio demonstrates a predictable and strong safety profile within the expected range reported in the broader literature, noting the limitations of cross-study comparisons. Most AEs were related to injection trauma rather than the filler itself, supporting its continued use in clinical aesthetic practice.

## 1. Introduction

The use of injectable dermal fillers has become a cornerstone of minimally invasive facial rejuvenation. Among the available filler classes, hyaluronic acid (HA)-based fillers remain the most widely used and extensively studied [1,2,3,4,5,6,7,8,9,10,11,12,13,14,15,16]. Their popularity stems from favorable characteristics such as high biocompatibility, hydrophilicity, and reversibility through enzymatic degradation with hyaluronidase [17,18,19,20,21,22,23,24,25,26,27]. Over the past two decades, a broad spectrum of HA fillers has emerged on the market, each designed with unique rheological properties tailored to specific aesthetic applications and anatomical regions. The primary indications for HA-based soft tissue fillers include the treatment of facial wrinkles and folds (e.g., nasolabial folds, marionette lines), volume restoration (e.g., cheeks, temples), contour enhancement (e.g., jawline and chin), tear trough correction, and lip augmentation. The adaptability of HA as a filler material is largely due to its ability to integrate into the dermal matrix, attract water, and provide both immediate and sustained volumizing effects. These features, along with a relatively low incidence of serious complications, have positioned HA fillers as a mainstay in aesthetic dermatology.

Despite their favorable risk-benefit profile, the use of HA fillers is not without complications [4,6,7,28,29,30,31,32]. Adverse events (AEs) can occur and should be understood as part of responsible clinical practice. Immediate or early-onset AEs typically include injection-site reactions such as erythema, edema, bruising, pain, and tenderness, which are usually mild to moderate and resolve spontaneously within a few days. Infrequently, early AEs may also include urticaria-like responses or mild localized infections. Delayed or long-term AEs, although less common, are clinically relevant due to their potential complexity and persistence. These include nodules, granulomatous inflammation, delayed hypersensitivity reactions, and biofilm-related infections. In a few cases, vascular complications such as intravascular injection, vascular occlusion, and tissue necrosis can occur, potentially leading to permanent sequelae, including skin loss or vision impairment if the filler enters the ophthalmic arterial system. Adverse events following HA filler treatments may be multifactorial and can be classified as procedure-related, patient-related, and product-related. Proper injection technique, correct product placement, and anatomical knowledge can reduce the incidence of procedure-related AEs. Furthermore, individual patient factors, such as a history of allergies, autoimmune diseases, concurrent medications, or other aesthetic procedures, may play a role in the development of adverse reactions. Long-term safety issues may also arise from the degradation behavior of the filler, persistence of the product in tissue, or immune responses triggered by filler constituents or impurities. The physicochemical characteristics of a filler significantly influence both its clinical performance and safety profile, including HA concentration, particle size, degree and type of cross-linking, and the presence of residual cross-linking agents. The cross-linking process is particularly critical, as unmodified HA is rapidly metabolized in vivo. Cross-linking introduces chemical bridges between HA chains, increasing durability and mechanical stability; however, excessive or irregular cross-linking or inconsistent manufacturing processes may contribute to immunogenicity, poor tissue integration, or late-onset complications. Therefore, the quality and control of the manufacturing process are vital in minimizing variability and ensuring product consistency.

This review specifically examines fillers manufactured using MACRO Core Technology because this crosslinking platform represents a distinct and unified manufacturing approach that allows for meaningful comparison of safety outcomes across the product portfolio. Evaluating fillers produced with the same technological backbone reduces heterogeneity related to differing manufacturing processes, BDDE content, particle size distribution, and purification methods—factors known to influence tolerability and adverse event profiles. By focusing on this coherent product family, the review aims to provide a scientifically consistent and clinically relevant assessment of safety that cannot be achieved by mixing data from unrelated HA filler technologies. Additionally, MACRO Core Technology fillers have undergone a recent expansion in clinical use, warranting an updated synthesis of their safety performance to support evidence-based decision-making in aesthetic practice [33,34,35,36,37].

## 2. Materials and Methods

**Figure 1:**
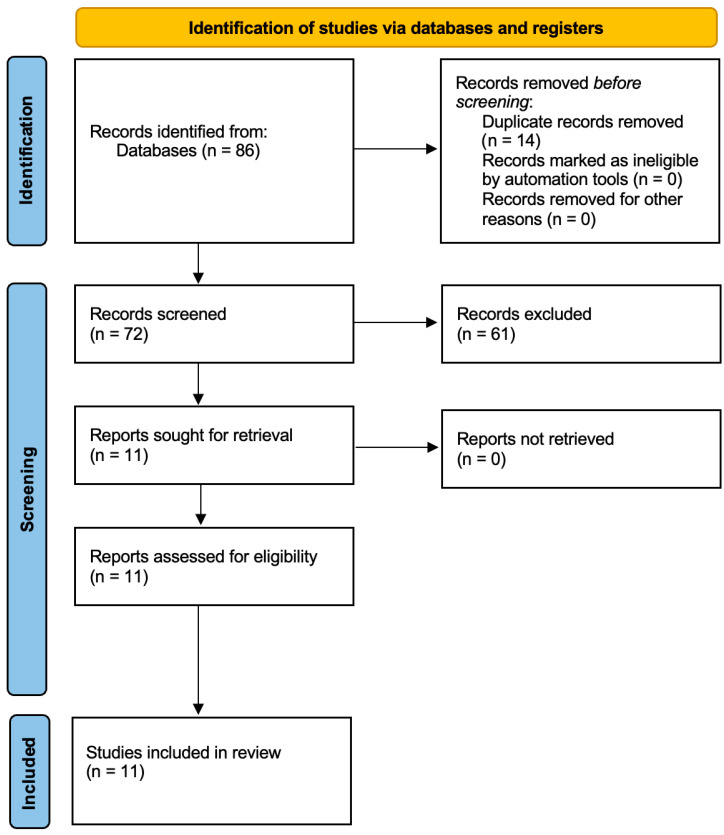
Flow diagram of the study selection process.

This systematic review was conducted and reported in accordance with the Preferred Reporting Items for Systematic Reviews and Meta-Analyses guidelines (PRISMA checklist in the Supplementary Materials). A structured and predefined methodology was applied for literature identification, screening, eligibility assessment, and data extraction to ensure transparency, reproducibility, and methodological rigor. The study selection process is summarized in the PRISMA flow diagram (Figure 1).

### 2.1. Eligibility Criteria

Clinical studies were included if they met the following criteria:

1. Reported on the safety of hyaluronic acid-based fillers within the saypha product range, including the products under former brand names Princess;
2. Involved the use of fillers for aesthetic facial indications (including, facial lipoatrophy, scarring, and asymmetry);
3. Reported on adverse events following filler injection;
4. Were conducted in human subjects;
5. Were published in English as clinical trials.

Exclusion criteria included studies involving other filler brands, non-clinical studies (e.g., in vitro or animal models), editorials, reviews, and studies reporting exclusively on efficacy without any safety outcomes. Studies were grouped by the filler product used and the nature of adverse events reported (short-term vs. long-term) to facilitate structured data synthesis.

### 2.2. Information Sources

The primary information source used for this review was PubMed, due to its comprehensive indexing of peer-reviewed biomedical and clinical literature. Reference lists of included articles were also screened manually to identify any additional eligible publications (Figure 1).

### 2.3. Search Strategy

The final PubMed search was conducted on 10 October 2025 using a combination of MeSH terms and free-text keywords to ensure comprehensive retrieval of relevant literature. The following search string was applied:

(“Saypha” OR “Princess Filler” OR “Princess Volume” OR “Princess Filler Lidocaine” OR “Saypha Filler” OR “Saypha Lips” OR “Saypha Volume” OR “Saypha Volume Plus”) AND (“hyaluronic acid” OR “HA filler” OR “dermal filler” OR “facial filler” OR “soft tissue filler” OR “injectable filler”) AND (“adverse effects” OR “adverse events” OR “complications” OR “side effects” OR “safety” OR “risk” OR “reaction” OR “inflammation” OR “nodule” OR “vascular” OR “granuloma”) AND (“aesthetic” OR “cosmetic” OR “rejuvenation” OR “facial contouring” OR “facial treatment” OR “beauty” OR “non-surgical”). No publication date restrictions were applied.

### 2.4. Selection Process

Two independent reviewers screened titles and abstracts for relevance. Full-text versions of potentially eligible articles were assessed using the predefined inclusion and exclusion criteria. Any disagreements were resolved through discussion or, where necessary, by consultation with a third reviewer. No automated tools or software were used during this process.

### 2.5. Data Collection Process

Data extraction was carried out independently by two reviewers using a standardized extraction template. The reviewers extracted study title, author(s), publication year, HA filler product used, aesthetic indication, number of participants, follow-up duration, number and type of adverse events, AE severity (mild, moderate, severe), and AE duration. Where severity classification was not explicitly stated, assumptions were made based on common clinical interpretations of the AE description.

### 2.6. Data Items

The primary outcome was the incidence and nature of adverse events associated with filler administration. Adverse events were classified as:

- Short-term: occurring within 30 days post-injection (e.g., swelling, erythema, pain, bruising);
- Long-term: occurring beyond 30 days (e.g., granulomatous inflammation, delayed-onset nodules, biofilm-related infections, hypersensitivity reactions).

Additional contextual data items included filler formulation details, injection technique (if reported), and other procedure-specific variables relevant to AE reporting.

### 2.7. Effect Measures

The primary measure was the number and percentage of patients experiencing one or more adverse events, reported as crude counts and proportions relative to the study population.

### 2.8. Synthesis Methods

Given the heterogeneity in study designs, injection techniques, and AE classification, no meta-analyses or statistical pooling of data were performed. Data were synthesized narratively and summarized in structured tables. No subgroup or sensitivity analyses were conducted.

### 2.9. Reporting Bias and Certainty Assessment

No formal tools were applied to assess publication bias or certainty of evidence. Only peer-reviewed clinical trials indexed in PubMed were included, and the consistency, transparency, and completeness of safety reporting across studies were used as qualitative markers of evidence reliability.

### 2.10. Risk of Bias Assessment

To strengthen methodological rigor, the risk of bias of each included prospective, non-randomized study was assessed using the ROBINS-I framework. Two independent reviewers evaluated the studies across seven domains (confounding, participant selection, intervention classification, deviations from intended interventions, missing data, outcome measurement, and selection of reported results). Discrepancies were resolved by consensus. The results of this assessment are presented in Table 1.

Table 1 Risk of bias assessment of included studies using the ROBINS-I tool. This table summarizes the risk of bias for each included prospective clinical study evaluating the safety of hyaluronic acid fillers within the saypha/Princess product portfolio. The ROBINS-I framework evaluates bias across seven domains: confounding, selection of participants, classification of interventions, deviations from intended interventions, missing data, outcome measurement, and selection of reported results. Because most included studies were non-randomized, open-label, and lacked blinded adverse event (AE) assessment, the overall risk of bias was generally rated as moderate. Higher risk ratings (moderate–serious) were assigned in studies with heterogeneous populations, limited long-term follow-up, or insufficient AE reporting detail. These assessments inform the interpretation of the body of evidence and were incorporated into the GRADE evaluation of certainty.

### 2.11. Certainty of Evidence (GRADE)

The certainty of the body of evidence for key safety outcomes was determined using the GRADE approach. Because most studies were non-randomized and open-label, evidence began at ‘low’ certainty and was further downgraded or upgraded based on risk of bias, inconsistency, indirectness, imprecision, and publication bias. Certainty ratings for each outcome are presented in Table 2.

This table presents the key safety outcomes from 11 prospective clinical trials involving 947 participants treated with hyaluronic acid fillers manufactured using MACRO technology. Outcomes include short-term adverse events (AEs ≤ 30 days), serious or severe device-related AEs, long-term complications (>30 days), vascular events, and withdrawals due to AEs. The GRADE framework was used to assess the overall certainty of evidence for each outcome. Certainty was rated low for common short-term AEs due to methodological limitations, heterogeneity in AE definitions, and unblinded assessments. Certainty for long-term or rare complications—including delayed-onset nodules, granulomas, and vascular events—was rated very low, reflecting limited sample sizes, incomplete long-term follow-up, and the inherent difficulty of detecting rare events in pre-market clinical trials. These ratings highlight that, while observed AE rates were generally low, the true incidence of rare or delayed complications remains uncertain and may differ in real-world settings.

## 3. Results

Each of the 11 included clinical studies assessed adverse events (AEs) following injection of hyaluronic acid fillers from the investigational range (Table 3).

AE profiles were characterized by treatment indication, filler formulation, number of affected subjects, severity of events, and their temporal resolution. Downie et al. conducted a randomized, multicenter split-face study on 270 subjects aimed to compare effectiveness and safety of PFL and Juvéderm Ultra XC (JUXC). A total of 66 patients (24.4%) reported treatment-emergent adverse events (TEAEs), with 21 patients (7.8%) experiencing TEAEs related to the procedure in PFL group. The most common events included headache (2.2%), swelling (1.5%), and contusions (1.5%). One serious device-related adverse event (0.4%) occurred. Notably, 243 patients (95.3%) experienced injection-site reactions (ISRs), of which 13.7% were classified as severe. Firmness, swelling, lumps, and bruising were frequent, particularly among those reporting severe ISRs. Moreover apart from confirmed noninferiority of PFL vs. JUXC, this study confirmed comparable safety profile for both products.

Dai et al. conducted a bilateral NLF treatment study in 120 Chinese subjects using PV on one side and Restylane (RL) on the other. AEs were reported in 58 patients (48.3%) for PV, most frequently swelling (30.8%), pain (21.7%), and nodules (16.7%). The incidence rates of AEs were similar after PV and RL treatments. Nearly all events were mild in intensity (99.0%) and resolved within 14 days. No severe AEs occurred. Grablowitz et al. evaluated 60 subjects treated with PFL and AEs were reported in 28 patients (46.7%), with 31 of 38 events (81.6%) classified as procedure-related rather than device-related. The most common AEs were injection-site hematoma (26.7%), pain (20%), and swelling (3.3%). All events were mild to moderate, with one hematoma resolving over 25 days. Two unrelated serious AEs were observed. In an earlier multicenter study by Kopera et al., 48 patients received PV for NLF correction. A total of 24 AEs occurred in 15 subjects (31.3%), with 12 hematomas and 2 swelling episodes being the most common. Two events (uterine polyp and trigger finger) were severe, but unrelated to the filler. Most AEs resolved by study end, and 66.7% were classified as mild.

A post-marketing study by Kopera et al. on PVL included 62 subjects and reported 18 AEs in 12 (19.4%) patients. Injection site hypoesthesia (9.7%) and pain (3.2%) were most common. No serious AEs were observed, and all events were mild, with resolution by study conclusion. A prospective study by Kopera et al. involved 53 patients treated with PF for facial lipoatrophy, scarring and asymmetry. 11 patients (21.0%) reported AEs, with five events (9.0%) considered treatment-related. These included mild hematomas and moderate injection site pain. All resolved within 3 weeks. No long-term complications or serious AEs were recorded.

The PRIMAvera study by Rzany et al. examined PVPL in 91 patients undergoing midface augmentation over 52 weeks. 73 AEs (43%) and 92 adverse device events (ADEs; 55%) were reported. Common ADEs included hematoma (37%), pain (23%), swelling (10%), and nodules (8%). All resolved within 85 days. Five serious AEs were recorded but deemed unrelated to treatment.

Mueller et al. conducted a prospective study in 114 patients receiving SLL. A total of 192 AEs were recorded, affecting 67 patients (58.8%). None were considered device-related; however, ≈75% of all AEs (n = 142) were linked to the injection procedure. The most frequent events included pain (41.3%), swelling (29.8%), and bruising (29%). Only one serious AE (disc prolapse) occurred and was unrelated to the filler or procedure. AE rates were higher with bolus vs. retrograde technique, but unaffected by equipment (needle vs. cannula).

In a study of 30 patients, Mueller et al. evaluated jawline augmentation with SVPL. AEs occurred in 23 subjects (77%), including bruising (16 cases), mastication disorder (11), swelling (10), and jaw pain (7). One AE was severe (disc herniation), but unrelated to treatment. Overall, 41 AEs were mild, 13 moderate, and most resolved during follow-up.

Another study by Mueller et al. evaluated SVPL in temple augmentation in 30 women. A total of 31 AEs were observed in 13 patients (43%). The most common events were bruising (6 patients), swelling (8), pain (7), and mastication disorder (6). Most resolved within 5–11 days, except one case of neuralgia, which resolved in 32 days. All events were mild or moderate, and no serious complications occurred.

In a study by Sulovsky et al., 59 female subjects received non-crosslinked HA filler for perioral and lateral canthal lines. 25 AEs were reported in 18 patients (31%), of which 84% were mild and 16% moderate. Most were procedure-related, including bruising (68%), oral herpes (4%), and swelling (4%). No severe or serious AEs were recorded.

### 3.1. Short-Term Adverse Events

Short-term adverse events (AEs), defined as those occurring within 30 days post-injection, were reported across all 11 included studies and typically developed within hours to days following treatment. The most commonly observed AEs in this timeframe were swelling, injection-site pain, bruising, erythema, firmness, and tenderness. Swelling emerged as the most frequent short-term AE, affecting 30.8% of participants (n = 37) in the study by Dai et al. for PV, 29.8% (n = 34) in the study by Mueller et al. on SLL, 25.0% (n = 12) in the study by Kopera et al. evaluating PV for nasolabial fold (NLF) correction, 9,9% in the study by Rzany et al. for PVPL and between 1.5% and 3.3% in other studies such as those by Downie et al. and Grablowitz et al.

Pain at the injection site was similarly prevalent. It was reported in 21.7% of subjects (n = 26) in the Dai et al. study, in 41.2% of subjects (n = 47) in the Mueller et al. SLL cohort, in 20.0% of patients (n = 12) in the study by Grablowitz et al., and in 23% of participants (n = 7) in Mueller et al.’s study on temple volumization. Bruising or hematoma formation was another frequent reaction. It occurred in 26.7% of patients (n = 16) in Grablowitz et al., in 25% (n = 12) in Kopera et al. (NLFs), in 16 patients during Mueller et al.’s jawline contouring study, and in as many as 68% of patients in the study by Sulovsky et al., which examined perioral and lateral canthal line correction. Additional transient reactions included erythema, tenderness, nodules—typically mild and self-resolving—and hypoesthesia, which was particularly notable at 9.7% in the post-marketing PVL study by Kopera et al. The majority of these short-term events were mild to moderate in severity and resolved spontaneously within 14 days. Only a small number of cases exceeded this duration; for example, one hematoma reported in Grablowitz et al. persisted for 25 days before resolution.

### 3.2. Long-Term Adverse Events

Long-term adverse events, defined as those occurring beyond 30 days post-injection, were rare among the included studies. One of the few notable long-term complications was the occurrence of delayed-onset nodules in 16.7% of patients in the Dai et al. study, although the timing of onset was not consistently reported, and the classification as long-term was made conservatively. Importantly, no instances of granulomatous inflammation, biofilm-related infections, or hypersensitivity reactions were documented in any of the reviewed studies.

In the study by Mueller et al., a case of neuralgia persisted for 32 days, representing the longest duration of any AE across all studies. However, this event was classified as moderate and resolved fully without further complications. Similarly, in the PRIMAvera study by Rzany et al., five serious adverse events (5.5%) were recorded; none were considered related to the filler or procedure. All adverse device events in that study resolved by day 85.

Overall, while local injection-site reactions were common and generally self-limited, no persistent, immunogenic, or life-threatening complications were observed in any of the included prospective clinical trials.

### 3.3. Results of Syntheses

Given the heterogeneity in product type, patient populations, treatment indications, and adverse event (AE) reporting methods across the included studies, a formal meta-analysis was not conducted. However, descriptive synthesis of the data revealed a consistent and predictable short-term AE profile across all filler products, primarily attributable to the mechanical effects of injection. Mild-to-moderate events such as swelling, bruising, and injection-site pain were the most frequently reported and generally resolved without intervention. The overall rate of serious or severe AEs was low, with only isolated cases documented across the studies, none of which were definitively linked to the filler products.

### 3.4. Risk of Bias Across Studies

ROBINS-I assessment showed that most studies had a moderate risk of bias, primarily related to the absence of randomization, potential confounding, heterogeneous AE definitions, and non-blinded outcome assessment. Several studies demonstrated unclear or higher risk in selective reporting domains due to limited detail on AE severity or onset. The complete ROBINS-I evaluation is provided in Table 1.

### 3.5. Certainty of Evidence (GRADE)

Using GRADE, the certainty of evidence for common short-term AEs was judged to be low, reflecting methodological limitations and inconsistency in AE classification. Certainty for long-term or rare complications was very low, largely due to sparse data, limited follow-up duration, and imprecision around rare event estimates. The Summary of Findings table (Table 2) provides detailed ratings.

Table 4 summarizes adverse events across the included saypha/Princess HA filler studies as a quick companion to Table 3. It reports, per study, the product/indication, sample size, total AEs, ISR counts (when available), severe events, and typical resolution time, plus a standardized AEs per 100 subjects metric to support easy cross-study comparison despite heterogeneous reporting. “NR” indicates outcomes not reported in the source study.

## 4. Discussion

This systematic review evaluated the safety profile of a portfolio of hyaluronic acid (HA)-based soft tissue fillers from the investigational range (Saypha and legacy Princess fillers), manufactured using MACRO Core Technology, with a focus on adverse events (AEs) reported in prospective clinical trials across aesthetic facial indications. Across 11 included studies (947 treated subjects), the overall safety profile was favorable and consistent, characterized predominantly by short-term, self-limiting injection-site reactions (e.g., swelling, bruising, pain, erythema, firmness, and transient nodules). Serious device-related events were rare. Overall, these findings align with the broader HA filler literature, which consistently reports that most AEs are mild to moderate local reactions resolving spontaneously within days to weeks. 

Short-term AEs were common but predictable and clinically manageable. Most occurred within the first 30 days post-injection and likely reflect expected mechanical and inflammatory responses to intradermal or subdermal HA filler placement. Frequently reported events included swelling, injection-site pain, erythema, bruising, nodules, and firmness. In most cases, these reactions resolved within 7–14 days without medical intervention, with a minority persisting up to 25–32 days. The incidence and nature of short-term AEs appeared broadly consistent across formulations with different predefined visco-elastic parameters, suggesting a stable safety profile across target anatomical areas, indications, and demographic differences. 

When contextualized against published syntheses, the observed AE pattern remains within the expected range for HA fillers. Kyriazidis et al. reported that HA dermal fillers are generally safe and effective, with most AEs transient and mild to moderate and severe AEs rare [25]. Colon et al., in a systematic review and meta-analysis of complications after HA-based injections, identified swelling as the most frequent AE (pooled incidence ~40.7%) and also highlighted pain, erythema, bruising, lumps/bumps, firmness, and tenderness as common reactions [49]. In the present dataset, swelling rates after treatment with Saypha and legacy Princess fillers ranged roughly from 1.5% to 33% depending on indication, product, and reporting framework. While this may suggest that swelling for the investigated products is within—and in some settings below—the range reported for other HA fillers, cross-study comparisons must be interpreted cautiously due to differences in design, patient selection, anatomical targets, injection techniques, AE definitions, and follow-up durations. This review was not designed or powered to support formal comparative claims, and the evidence does not justify concluding clinical superiority regarding swelling or other short-term AEs. 

Real-world evidence (RWE) from large cohorts complements these trial-based observations and underscores that HA fillers, as a class, have an acceptable safety profile but are not devoid of risk. Nishikawa et al. reported early complications across 41,775 HA filler procedures and found complications were uncommon and mostly manageable, though ischemic events and necrosis occurred in a small minority. Tamura et al. reviewed 290,307 injections and identified 12 serious complications (primarily infections and vascular obstructions), illustrating that while severe AEs are rare, the absolute number of affected patients can be clinically meaningful as HA filler use expands. In this context, the AE profile observed in the included Saypha and legacy Princess trials falls within the established safety range for HA fillers overall. 

Long-term or delayed AEs were infrequently reported in the included trials. Nodules were described in one study with an incidence of 16.7%, although timing of onset was not systematically captured; in another study evaluating the same product for nasolabial folds, no nodules were observed. Nodules were also reported in one additional study investigating PVPL at an incidence of 8%, slightly lower than the 9.5% reported by Colon et al. for comparable products. None of the trials reported granulomatous inflammation, delayed hypersensitivity reactions, biofilm-related infections, or immune-mediated inflammatory events. While reassuring, these findings should be interpreted with care because clinical trials—by virtue of controlled inclusion criteria, intensive monitoring, homogeneous populations, and limited size/duration—are generally underpowered to detect rare or late-onset complications more likely to emerge in routine practice or post-market surveillance. 

Serious device-related AEs were rare. Only one serious adverse device effect was reported (a treatment-emergent adverse device effect classified as serious and unanticipated) in a study of Princess Filler Lidocaine, occurring in 1 of 270 patients (0.4%). Other serious AEs recorded in the included studies (e.g., thyroid cancer, uterine polyps, vertebral disc herniation) were judged unrelated to the device or procedure. No vascular occlusions, skin necrosis, or vision-threatening events were reported in the included trials—consistent with the broader literature in which such severe events are rare but documented primarily through case reports, registries, and post-market surveillance rather than pre-market clinical trials. 

The manufacturing platform (MACRO Core Technology) may plausibly contribute to safety and tolerability, though causality cannot be established from the included evidence. MACRO technology involves controlled crosslinking with 1,4-butanediol diglycidyl ether (BDDE), incorporated as double bonds between high molecular weight HA chains to create an efficient cross-linked network while minimizing overall BDDE content. Unbound BDDE and other potential impurities are removed through multi-step purification (including repeated filtration, dialysis, and AeroVac purification to remove air inclusions), supporting purity, stability, and biocompatibility. Puljic et al. reported that Macro Core Technology shows a narrow particle size range (DV50 351–374 µm) compared with other HA fillers manufactured with different crosslinking technologies. Rather than increasing particle size, MACRO adjusts gel content and crosslinking degree to create a rheologically versatile (G’) portfolio. Small particles, narrow particle-size distribution, and large-scale mixing of cross-linked and non-cross-linked HA phases yield a homogeneous product with batch-to-batch consistency. These features may help reduce risks such as granulomatous inflammation or immunogenicity, which have been associated in the literature with impurities or inconsistencies in gel composition; however, the studies in this review were not designed to evaluate MACRO technology as a determinant of safety, and cross-study comparisons cannot support causal attribution. 

Several limitations constrain interpretation. Across the included trials, heterogeneity in study design, indication, sample size, follow-up duration, and AE classification frameworks complicates cross-study comparability and precludes meaningful meta-analysis. Some studies report AEs per patient while others report per injection site; severity scales and time windows differ; and the distinction between device-, procedure-, and patient-related AEs is not applied uniformly—introducing uncertainty in incidence estimates and reinforcing the appropriateness of a structured narrative synthesis. The absence of standardized AE definitions across publications likely contributes to reporting variability. The evidence base is further qualified by formal risk-of-bias assessment: ROBINS-I ratings were mostly moderate, driven by non-randomized and open-label designs, limited blinding for outcome assessment, and heterogeneity in AE reporting—meaning that while the overall AE pattern supports a strong short-term safety profile, the available data do not permit highly precise risk estimates. 

Additional constraints include the reliance on PubMed-indexed prospective clinical trials, which may exclude relevant real-world evidence or older studies (including those using earlier product nomenclature). Publication bias cannot be ruled out, as studies with favorable safety outcomes may be more likely to be published. Importantly, all included studies were manufacturer-involved (company-sponsored regulatory/post-market studies) or investigator-initiated but focused exclusively on products from the same company; this sponsorship context, combined with exclusion of RWE sources such as registries, observational cohorts, and case series, likely under-represents rare, delayed, or off-label complications (e.g., delayed-onset inflammatory nodules, granulomas, and vascular events in higher-risk anatomical regions) that are more commonly captured outside tightly controlled trials. Consequently, the present analysis may present an optimistic safety view relative to routine clinical practice, particularly regarding very rare or delayed events. These limitations collectively highlight the need for standardized AE definitions and reporting guidelines specific to aesthetic injectables and for integration of high-quality RWE alongside trial data.

## 5. Conclusions

This review demonstrates that saypha and legacy Princess HA fillers exhibit a consistent short-term safety profile, with most reported adverse events being mild, injection-related, and self-limiting. The available data suggest that the incidence and nature of these events are comparable to, or in some cases lower than, rates reported for other HA fillers in the existing literature. At the same time, formal ROBINS-I and GRADE assessments indicate that the overall certainty of the evidence supporting these findings is *low*, largely due to methodological limitations, heterogeneous reporting, and limited follow-up durations across studies. Within these constraints, the incorporation of MACRO Core Technology may contribute to product consistency and purity; however, conclusions regarding its impact on clinical safety must remain cautious. Larger, independently conducted studies using standardized adverse event definitions and extended follow-up are needed to more definitively characterize the long-term safety profile of these products in real-world practice.

## Figures and Tables

**Table 1:** ROBINS-I table.

Study	Confounding	Selection	Classification	Deviations	Missing Data	Outcome Measurement	Selective Reporting	Overall Risk
Downie 2025 [38]	M	L	L	L	L	M	M	M
Dai 2019 [39]	M	L	L	L	L	M	M	M
Grablowitz 2019 [40]	M	L	L	L	L	M	M	M
Kopera 2015 [41]	M	L	L	L	M	M	M	M–S
Kopera 2020 [42]	M	L	L	L	L–M	M	M	M
Kopera 2018 [43]	M	L	L	L–M	M	M	M	M–S
Müller 2024 [44]	M	L	L	L	L	M	M	M
Müller 2022 [45]	M	L	L	L	L–M	M	M	M–S
Müller 2021 [46]	M	L	L	L	L–M	M	M	M
Rzany 2024 [47]	M	L	L	L	L–M	M	M	M
Sulovsky 2022 [48]	M	L	L	L	L–M	M	M	M–S

**Table 2:** Summary of findings and certainty of evidence for safety outcomes associated with saypha/Princess hyaluronic acid fillers (GRADE table).

Outcome	Participants	Follow-Up	Design	Findings	Certainty	Comments
Any short-term AE	947 (11 studies)	14–85 days	Prospective trials	20–80% mild/moderate AEs	Low	Risk of bias & inconsistency
Serious/severe device-related AEs	947	≤3 months	Prospective trials	1 serious AE (0.4%)	Low	Rare events, imprecision
Long-term complications	947	Up to 52 wks	Prospective trials	Nodules 8–16.7%, no granulomas	Very low	Sparse data, short FU
Vascular complications	947	Up to 52 wks	Prospective trials	No events reported	Very low	Rare; trials underpowered
Withdrawal due to AE	947	Up to 52 wks	Prospective trials	Very rare or absent	Low	Sparse data

**Table 3:** Summary of Included Clinical Studies Evaluating Safety Outcomes of MACRO Core Technology–Based HA Fillers.

Authors	Year of Publication	Title	Filler Product Used	Indication	Subjects Included	Number of AEs	Number of ISRs	Mild AEs	Moderate AEs	Severe AEs	Duration
Downie et al. [38]	2025	Multicenter, Randomized Split-Face Trial of a Crosslinked Hyaluronic Acid Fillers With Lidocaine for Nasolabial Fold Correction	Saypha Filler Lidocaine (SFL)	Nasolabial folds	270	66	243	43 (15.9%)	18 (6.7%)	5 (1.9%)	1–14 days; 3 cases > 14 days
Dai et al. [39]	2019	Safety and effectiveness of hyaluronic acid dermal filler in correction of moderate-to-severe nasolabial folds in Chinese subjects	Princess Volume (PV)	Nasolabial folds	120	58	Not specified	Not specified	Not specified	0	≤14 days in 98.28%
Grablowitz et al. [40]	2019	Safety And Efficacy Of Princess^®^ FILLER Lidocaine In The Correction Of Nasolabial Folds	Princess Filler Lidocaine (SFL)	Nasolabial folds	60	38	All at injection site	Majority	Few	0	≤14 days; one hematoma 25 days
Kopera et al. [41]	2015	An Open-Label Uncontrolled, Multicenter Study for the Evaluation of the Efficacy and Safety of the Dermal Filler Princess VOLUME in the Treatment of Nasolabial Folds	Princess Volume	Nasolabial folds	48	24	14	16	6	2	Most resolved by study end
Kopera et al. [42]	2020	A prospective, open label, multicenter, post market study evaluating Princess^®^ VOLUME Lidocaine for the correction of nasolabial folds	Princess Volume Lidocaine (PVL)	Nasolabial folds	62	18	9	All	0	0	Resolved by study end
Kopera et al. [43]	2018	Treatment of facial lipoatrophy, morphological asymmetry, or debilitating scars with the hyaluronic acid dermal filler Princess^®^ FILLER	Princess Filler	Facial lipoatrophy, morphological asymmetry, scars	53	11	Not specified	3	2	0	2–21 days
Mueller et al. [44]	2024	Lip Augmentation With Saypha LIPS Lidocaine: A Postmarket, Prospective, Open-Label, Randomized Clinical Study To Evaluate Its Efficacy and Short- and Long-term Safety	Saypha Lips Lidocaine (SLL)	Lip augmentation	114	192	124	Most	Some	1	Resolved within study
Mueller et al. [45]	2021	Longevity and subject-reported satisfaction after minimally invasive jawline contouring	Saypha Volume Plus Lidocaine (SVPL)	Jawline contouring	30	55	Not specified	41	13	1	Most resolved during FUP
Mueller et al. [46]	2021	Volumization of the young and the old temple using a highly cross-linked HA filler	Saypha Volume Plus Lidocaine (SVPL)	Temple volumization	30	31	Not specified	Most	Few	0	5–32 days
Rzany et al. [47]	2023	Long-term Performance and Safety of Princess VOLUME PLUS Lidocaine for Midface Augmentation: The PRIMAvera Clinical Study	Princess Volume Plus Lidocaine (SVPL)	Midface augmentation	91	73	92	Most	Few	5	Resolved ≤85 days
Sulovsky et al. [48]	2021	A prospective open-label, multicentre study evaluating a non-cross- linked hyaluronic acid based soft-tissue filler in the correction of lateral canthal and perioral lines	Saypha Rich (SR)	Lateral canthal and perioral lines	59	25	Not specified	21	4	0	Most ≤7 days

**Table 4:** Concise summary table of findings.

Study (Author, Year)	Product	Indication	N	AEs (n)	AEs per 100 Subjects	ISRs (n)	Severe AEs (n)	Typical Duration/Resolution
Downie et al., 2025 [38]	SFL	Nasolabial folds	270	66	24.4	243	5 (1.9%)	1–14 days; 3 cases > 14 days
Dai et al., 2019 [39]	PV	Nasolabial folds	120	58	48.3	Not reported	0	≤14 days in 98.28%
Grablowitz et al., 2019 [40]	SFL	Nasolabial folds	60	38	63.3	Not reported	0	≤14 days; one hematoma 25 days
Kopera et al., 2015 [41]	PV	Nasolabial folds	48	24	50.0	14	2	Most resolved by study end
Kopera et al., 2020 [42]	PVL	Nasolabial folds	62	18	29.0	9	0	Resolved by study end
Kopera et al., 2018 [43]	Princess Filler	Lipoatrophy/asymmetry/scars	53	11	20.8	NR	0	2–21 days
Mueller et al., 2024 [44]	SLL	Lip augmentation	114	192	168.4	124	1	Resolved within study
Mueller et al., 2021 [45]	SVPL	Jawline contouring	30	55	183.3	NR	1	Most resolved during follow-up
Mueller et al., 2021 [46]	SVPL	Temple volumization	30	31	103.3	NR	0	5–32 days
Rzany et al., 2023 [47]	SVPL	Midface augmentation	91	73	80.2	92	5	Resolved ≤85 days
Sulovsky et al., 2021 [48]	SR	Lateral canthal/perioral lines	59	25	42.4	NR	0	Most ≤7 days

## Data Availability

The original data presented in the study are openly available.

## Supplementary Materials

The following supporting information can be downloaded at: https://www.mdpi.com/article/10.3390/life16010110/s1, Table S1: PPRISMA checklist.
